# The Longitudinal Influence of Parent–Grandparent Coparenting Relationships on Preschoolers’ Eating Behaviors in Chinese Urban Families: The Mediating Roles of Caregivers’ Feeding Behaviors

**DOI:** 10.3390/nu17182961

**Published:** 2025-09-15

**Authors:** Zhihui Zhao, Fangge Qu, Ruxing Wu, Xiaoxue Wei, Xinyi Song, Chenjun Wu, Jian Wang, Wenzhe Hua, Daqiao Zhu

**Affiliations:** 1 School of Nursing, Shanghai Jiao Tong University, Shanghai 200025, China; stjuzzh527@sjtu.edu.cn (Z.Z.); qufangge@126.com (F.Q.); wu_ruxing@163.com (R.W.); songxinyi@sjtu.edu.cn (X.S.); wuchenjun0634@163.com (C.W.); 2 School of Nursing and Health Management, Shanghai University of Medicine & Health Sciences, Shanghai 201318, China; 3 Department of Hematology and Oncology, Shanghai Children’s Medical Center Affiliated to Shanghai Jiao Tong University School of Medicine, Shanghai 200127, China; weixiaoxue1998@163.com; 4 Florence Nightingale Faculty of Nursing, Midwifery and Palliative Care, King’s College London, London SE1 8WA, UK; k21087857@kcl.ac.uk

**Keywords:** parent–grandparent coparenting, caregivers, preschool children, eating behaviors, feeding behaviors

## Abstract

Background: The early development of children’s eating behaviors is a complex process shaped by dynamic interactions within the family system. While extensive research has focused on parental feeding practices as a primary predictor of children’s eating behaviors, the role of intergenerational coparenting dynamics (especially with involved grandparents) is less studied. This study aimed to examines how primary caregivers’ feeding behaviors mediate the relationship between parent–grandparent coparenting dynamics and children’s eating behaviors. Methods: We conducted a longitudinal study among 343 stem families with preschool children. The cross-lagged analysis was applied to examine: the mechanism of parent–grandparent coparenting relationships on preschool children’s eating behaviors and the bidirectional relationship between feeding behaviors and eating behaviors. Results: Our results revealed that coparenting agreement significantly and negatively predicted satiety responsiveness through the mediating role of parental encouragement of healthy eating (*β* = −0.012, 95% CI [−0.022, −0.001]). Similarly, coparenting support had a significant negatively indirect effect on satiety responsiveness, also partially mediated by parental encouragement of healthy eating (*β* = −0.012, 95% CI [−0.023, −0.002]). The association between coparenting undermining and satiety responsiveness was mediated by parental encouragement healthy eating (*β* = −0.612, 95% CI [−0.796, −0.429]). Mutual predictive relationships were observed between preschool children’s satiety responsiveness and parental encouragement of healthy eating (*p* < 0.05). Conclusions: The study results highlighted the critical role of parent–grandparent coparenting relationships as indirect predictors of preschoolers’ eating behavior through mediator of parental feeding behaviors. Importantly, healthcare providers may be able to offer anticipatory guidance or relevant healthy eating resources to parents and grandparents, who should be recognized as important stakeholders in promoting healthy eating among children.

## 1. Introduction

The increasing involvement of grandparents as primary caregivers for their grandchildren is a growing global trend, driven by demographic shifts and evolving family structures [1]. This phenomenon is particularly evident in societies where women’s workforce participation is rising and populations are aging, necessitating greater reliance on intergenerational caregiving [2]. The concept of parent–grandparent coparenting, also referred to as intergenerational coparenting, extends from traditional coparenting frameworks and describes the collaborative dynamics between parents and grandparents in child-rearing [3]. This caregiving arrangement is prevalent across both Western and Eastern societies, though its underlying motivations differ.

In the United States, approximately one million children are raised solely by their grandparents without parental presence [4]. In urban China, nearly 40% of grandparents are actively engaged in their grandchildren’s upbringing [5]. Although, the coparenting dynamic involving parents and grandparents is a phenomenon present in both Eastern and Western societies [5]. The motivations behind grandparental involvement vary significantly between cultural contexts. In individualistic Western societies, where nuclear family structures are dominant, grandparental caregiving often emerges as a response to crises such as parental divorce, substance abuse, or neglect [6]. In contrast, collectivist societies like China, shaped by Confucian values, emphasize familial interdependence, and grandparental participation is widely regarded as an essential element of a harmonious family unit [7]. In Asian countries and regions such as China, grandparental involvement in childcare is regarded as a cultural expectation [8]. On one hand, with the accelerated pace of life and mounting life pressure in China, an increasing number of young parents have shifted the responsibility of raising young children to their elderly family members, and grandparental care has gradually taken on a significant role in early childhood family education. On the other hand, China has been a nation that places great emphasis on family values since ancient times. Elderly individuals with rich life experience have long been regarded as “significant others” in children’s education. The life of “enjoying the company of grandchildren” (a traditional Chinese concept depicting the joy of grandparents caring for their grandchildren) is viewed by the vast majority of grandparents as an ideal way of life in their later years, and it is also a responsibility that they believe they should consciously undertake. Additionally, more than four decades have passed since China implemented the one-child policy in 1979 [9], and many urban families have evolved into multi-generational households where three generations live together. It is evident from this that grandparental participation in early childhood care is common and has a long-standing history in Chinese families. To better understand the scope of intergenerational coparenting, previous research has classified grandparental involvement into three distinct categories: (a) custodial grandparents, who serve as primary caregivers; (b) co-resident grandparents, who share a household with their children and grandchildren in multigenerational living arrangements; and (c) non-co-resident grandparents, who remain actively involved despite living separately [8]. In this study, parent–grandparent coparenting is defined as the active involvement of grandparents in the daily caregiving and upbringing of their grandchildren [9]. Despite extensive research on these arrangements, their impact on children’s eating behaviors—a critical determinant of long-term health—remains underexplored, particularly in longitudinal frameworks [10].

### 1.1. Intergenerational Coparenting and Child Eating Behaviors

While much research has focused on the structural aspects of grandparental caregiving, a critical yet underexplored dimension is its impact on children’s eating behaviors. Recently, a qualitative meta-analysis has highlighted the significant role of grandparents in shaping children’s eating habits [10]. However, the long-term effects of intergenerational coparenting relationships on children’s eating behaviors remain unclear, particularly in a longitudinal context. This gap in the literature is especially concerning given the increasing prevalence of childhood obesity, which has become a global public health crisis. The preschool period refers to the developmental stage of children aged 3 to 6 years, prior to their enrollment in primary school. This stage is critical for the formation of children’s dietary and eating behavior habits, which, once established, can persist throughout an individual’s lifetime [11]. The impact of poor eating behavior during early childhood extends beyond physical health, influencing mental health, cognitive development, and emotional well-being as well [12]. According to the 2024 World Obesity Report, an estimated 159 million children worldwide are affected by obesity [13]. In China, national health reports indicate that 11.1% of children aged 6–17 years and 6.8% of children under six are classified as obese [14]. Diet is widely acknowledged as one of the most significant modifiable factors in the prevention of obesity [15]. Considering the high prevalence of childhood obesity, identifying and prioritizing the determinants that shape children’s dietary and eating behaviors becomes essential.

One of the most significant predictors of childhood obesity is early-life feeding practices, which shape long-term dietary habits [16,17,18]. Existing research categorizes parents’ feeding practices into two broad types: responsive feeding and non-responsive feeding. Responsive feeding—which includes structured practices such as role modeling healthy eating, monitoring intake, and encouraging balanced nutrition-is associated with ideal growth trajectories, optimal nutrient intake, and effective weight regulation [19]. In contrast, non-responsive feeding, characterized by coercive control strategies (e.g., pressure to eat, excessive restriction, and instrumental feeding), is linked to unhealthy eating behaviors and increased BMI-z scores in children [17,20,21]. However, the role of intergenerational coparenting in this bidirectional dynamic remains underexplored.

The family systems theory (FST) [22] and the Ecological Model of Coparenting [23], suggest that harmonious intergenerational coparenting relationships contribute to children’s social competence and behavioral development [5,7]. Family Systems Theory posits that the family constitutes an integrated system composed of all members and their interactions, encompassing multiple interdependent subsystems (e.g., parent–child, marital, and parental subsystems) [21]. The family serves as the environment where children engage most frequently with the parent–child subsystem, and the interaction scenarios within this parent–child subsystem are primarily associated with the feeding environment provided by parents and grandparents—including the feeding methods they adopt, as well as the selection of dining locations and dining environments [5]. On the one hand, positive intergenerational coparenting relationships contribute to maintaining the equilibrium of the family system, thereby benefiting child development [24]. On the other hand, interpersonal conflicts or disharmony between parents and grandparents in co-parenting arrangements may result in diminished attentiveness to the child, reduced sensitivity in responding to the child’s needs, and heightened emotional insecurity in the child, which could further manifest in behavioral problems [5]. To our knowledge, only several published qualitative studies have reported that children are more likely to develop eating behavior problems such as picky eating, partiality, and food refusal when there is conflict or inconsistency in the coparenting relationship [25,26]. Hence, it remains unclear how intergenerational coparenting relationships shape children’s eating behaviors over time.

### 1.2. Theoretical Framework and Hypotheses

**Figure 1:**
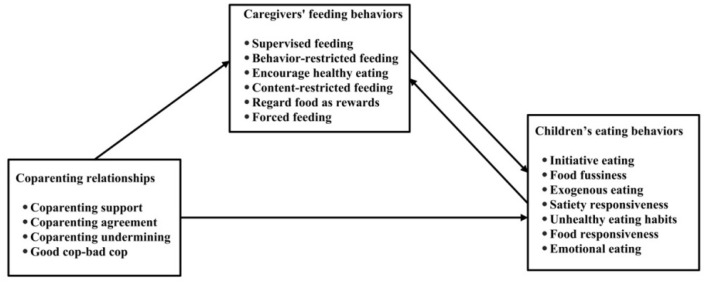
Conceptual framework of the study.

Grounding our study in the Ecological Model of Coparenting [23] and Family Systems Theory [22], we posit that harmonious parent–grandparent coparenting stabilizes family dynamics, fostering optimal child development [5]. Conversely, discord may reduce caregivers’ responsiveness to children’s needs, heightening emotional insecurity and maladaptive eating behaviors [5]. By integrating family systems theory with empirical feeding literature, we propose two hypotheses (Figure 1):

**H1:**  *The parent–grandparent coparenting relationship exerts (a) a direct influence on children’s eating behaviors and (b) an indirect influence mediated by caregivers’ feeding practices.*

Prior work demonstrates that coparenting quality predicts child social competence [7], but its dietary implications are untested. We extend this by hypothesizing that cooperative coparenting aligns caregivers’ feeding strategies (e.g., consistent meal routines), whereas conflict may lead to inconsistent practices (e.g., grandparents indulging unhealthy foods despite parental restrictions), indirectly affecting child eating habits.

**H2:**  *A bidirectional relationship exists between parental feeding practices and children’s eating behaviors.*

The existing evidence regarding the bidirectional association between parental feeding practices and children’s eating behaviors is inconsistent. A meta-analysis of fourteen longitudinal studies showed that parents using non-responsive feeding behaviors such as food as a reward was a positive predictor of children’s emotional eating [17]. Furthermore, the combined effect of this meta-study indicates that children’s satiety responsiveness negatively predicts parents’ restrictive feeding behavior [17]. Overall, Preschool years (3–6 years) are critical for establishing lifelong eating habits [16]. While parents are primary nutritional gatekeepers, grandparents increasingly act as secondary feeders [2]. This study employs longitudinal data to disentangle the interplay of intergenerational coparenting, feeding practices, and child eating behaviors, informing family-centered interventions to prevent childhood obesity by optimizing coparenting cohesion around nutrition.

## 2. Methods

### 2.1. Study Population

We recruited primary caregivers of 3–6-year-olds through a Pudong New Area community health center. All eight government-registered kindergartens in its jurisdiction meeting enrollment and participation criteria were included. The inclusion criteria were: (1) multigenerational households with grandparents, parents, and children, (2) families with preschool children aged 3 to 6 years, and (3) the primary feeders of children who participated in coparenting. The exclusion criteria were: (1) children with digestive system disease, inherited metabolic diseases, congenital diseases, or intellectual disabilities, attention deficit hyperactivity disorder (ADHD), (2) children who have suffered from various acute and chronic diseases in the past month, and their appetite has been affected, and (3) parents who are unwilling to participate in this study, unable to complete the questionnaire independently or have communication difficulties. These conditions were ascertained through structured parent-report questions in the screening questionnaire, with available kindergarten health records cross-referenced for verification where possible. All procedures involving human participants were approved by the Research Ethics Committee of Shanghai Jiao Tong University (No. SJUPN-201908).

**Figure 2:**
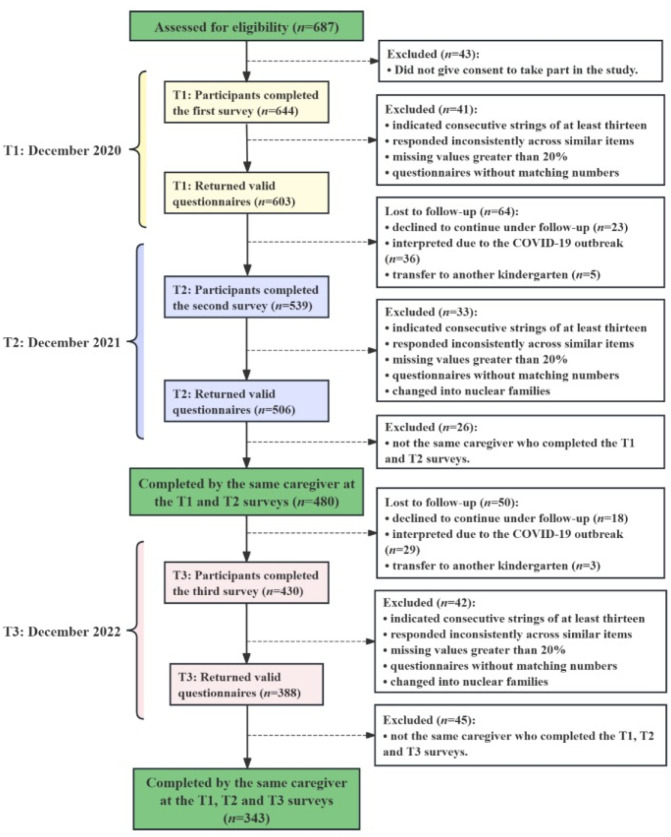
Flow chart of the number of screened potential participants, recruitment, and follow-up.

Longitudinal data were collected in December 2020 (T1), December 2021 (T2), and December 2022 (T3). Of the 687 parents who enrolled in the study, 388 completed three follow-up surveys. Of the 388 completed surveys, 343 were valid, resulting in a 88.4% effectiveness rating. Parents dropped out of follow-ups for the following main reasons: too busy to continue cooperating with the study (*n* = 41), the impact of COVID-19 (*n* = 65). The criteria for invalid questionnaires were: obvious regular answers (i.e., all items in a scale had the same answer), more than 20% missing values, and no identifier for matching. Participants were excluded if the surveys at the three-time points were not completed by the same caregiver. Therefore, the final sample consisted of 343 parents (Figure 2).

### 2.2. Procedure

Research staff distributed paper questionnaires through community health centers and kindergartens after obtaining written informed consent. Participants completed questionnaires independently at home and returned them within two weeks using prepaid envelopes. To ensure data reliability, kindergarten teachers provided reminder calls during the return period. Each participant received a unique ID for tracking across three waves (baseline, 6-month, 12-month follow-ups). To mitigate practice effects, questionnaire sections followed a fixed rotation sequence: Demographic/Coparenting (Section A), Feeding Practices (B), and Child Behaviors (C) were ordered as A-B-C at baseline, B-C-A at 6 months, and C-A-B at 12 months. Completed questionnaires were collected by research staff who verified ID matching before data entry.

### 2.3. Measures

#### 2.3.1. Sociodemographic Information

Sociodemographic information collected included: children’s characteristics (age, gender, single-child or not, duration of breastfeeding), the primary caregiver’s characteristics (identity, age, height, weight, education, annual family income), and the co-parenting caregivers’ characteristics (identity, age, height, weight, education, living together with children or not). These variables may influence coparenting relationships [27], feeding behaviors [28], and eating behaviors.

#### 2.3.2. Anthropometric Measurement

The BMI of adults was calculated by the formula: BMI = weight (kg)/height (m)^2^. According to the Guidelines for Prevention and Control of Overweight and Obesity in Chinese Adults [29], we classified adults’ BMI status as follows: underweight (BMI < 18.5 kg/m^2^), normal weight (18.5 kg/m^2^ ≤ BMI ≤ 23.9 kg/m^2^), overweight (24.0 kg/m^2^ ≤ BMI ≤ 27.9 kg/m^2^), and obese (BMI ≥ 28.0 kg/m^2^).

During each follow-up survey, children’s height and weight were measured by pediatricians or health teachers in accordance with the national standard of the People’s Republic of China [30]. Without shoes, hats, and heavy clothing, children stood upright on a calibrated anthropometric scale (0.1 cm precision in height and 0.01 kg precision in weight) with eyes straight forward, arms down, and feet together. BMI-for-age Z score (BAZ) was calculated using WHO Anthro software version 3.2.2 (<5 years) and WHO AnthroPlus software version 1.0.4 (≥5 years) based on children’s height, weight, gender, and age. According to the Child Growth Standards of the Word Heath Organization [31], we classified children’s BMI status as follows: underweight (BAZ < −2), normal weight (−2 ≤ BAZ ≤ 1), overweight (1 < BAZ ≤ 2), and obese (BAZ > 2).

#### 2.3.3. Parent–Grandparent Coparenting Relationships

The Grandparents-Parents Coparenting Relationships Scale (GPCRS) [32] was used to measure the following dimensions of coparenting between parents and grandparents: coparenting agreement (whether the views of how to rear a child are similar or not, 4 items), coparenting support (one’s perception of coparenting support from a partner, 6 items) and coparenting undermining (one’s criticism, disparagement, and blame of the other partner, 6 items).

Considering the uniqueness of the Chinese cultural background and the qualitative research foundation of our early studies, we added an extra dimension of measurement- ‘good cop-bad cop’, a typical coparenting relationship specific to Chinese families and integrated cooperation and conflict during coparenting, similar to the model of “strict father and loving mother” in Chinese culture. The dimension of ‘good cop-bad cop’ consists of five items measured with the Coparenting Scale (C.S.) in Chinese families revised by Wang et al. [33].

All items were scored on a 7-point Likert-type response scale ranging from “not true of us” (0) to “very true of us” (6). The scores of each dimension were the mean value of items (0–6), with higher scores indicating a greater frequency of behaviors on that particular dimension. The coparenting agreement and coparenting support subscales measure the positive aspect of the co-parenting relationships, with higher scores indicating higher quality of parent–grandparent coparenting relationships. The coparenting undermining and good cop-bad cop subscales measure the negative aspect of the co-parenting relationships, with higher scores indicating more parent–grandparent conflict during coparenting. In this study, the internal consistency reliability of each subscale evaluated by Cronbach’s alpha was 0.745 (coparenting agreement)–0.863 (good cop-bad cop) at T1, 0.724 (coparenting agreement)–0.894 (good cop-bad cop) at T2, and 0.746 (coparenting agreement)–0.855 (good cop-bad cop) at T3.

#### 2.3.4. Caregivers’ Feeding Behaviors

The Chinese Preschoolers’ Caregivers’ Feeding Behavior Scale (CPCFBS) developed by Yuan et al. [34]. was used to evaluate five types of caregivers’ feeding practices, including content-restricted feeding (strict limitations on the children’s access to foods or opportunities to consume unhealthy foods, 4 items), behavior-restricted feeding (modeling of healthy food choice to encourage children to adopt similar behaviors, 7 items), encourage healthy eating (the behaviors of encouraging their children to eat more healthy food, 6 items), forced feeding (insists, demands, or physically struggles with the child in order to get the child to eat more food, 3 items) and supervise eating (the extent to oversee their child eating, 4 items). Among the five types of feeding behaviors measured, force-feeding was non-responsive feeding behavior, while the others were responsive feeding behaviors.

All items were scored on a 5-point Likert-type response scale ranging from “never” (1) to “always” (5). The scores of each dimension were the mean value of items (1–5), with higher scores indicating a higher frequency of feeding practices. In this study, the internal consistency reliability of each subscale evaluated by Cronbach’s alpha was 0.712 (encourage healthy eating)–0.883 (supervise eating) at T1, 0.743 (forced feeding)–0.897 (supervise eating) at T2, and 0.767 (forced feeding)–0.886 (supervise eating) at T3.

#### 2.3.5. Preschool Children’s Eating Behaviors

Parents filled out the Chinese Preschoolers’ Eating Behavior Questionnaire (CPEBQ) developed by Zhang et al. [35], which measures preschool children’s eating behaviors. This is a questionnaire completed by parents about their children including unhealthy eating habits (watching T.V. or playing with toys during meals, keeping food in the mouth for a long time without swallowing it, etc., 4 items), food fussiness (reluctance to try new food or eating limited food, 5 items), emotional eating (eating in response to happiness, anger, worry, sadness, etc., 5 items), satiety responsiveness (the limited amount of food the child eats in a meal, 5 items), food responsiveness (the desire to eat food when children see or smell food, or were supplied with food, 6 items), exogenous eating (the influence of external factors, such as dishware, food kind, eating environment, other people’s eating behaviors, etc., 5 items) and initiated eating (the ability of child independent eating, 5 items).

All items were scored on a 5-point Likert-type response scale ranging from “never” (1) to “always” (5). The scores of each dimension were the mean value of items (1–5), with higher scores indicating a higher frequency of a particular type of eating behavior. In this study, the internal consistency reliability of each subscale evaluated by Cronbach’s alpha was 0.681 (initiated eating)–0.770 (satiety responsiveness) at T1, 0.598 (exogenous eating)–0.826 (emotional eating) at T2, and 0.616 (exogenous eating)–0.793 (emotional eating) at T3.

### 2.4. Statistical Analysis

Statistical analyses and mapping were performed using Microsoft Excel (Office 2019), SPSS (Version 24.0), and Mplus (Version 8.3).

**Figure 3:**
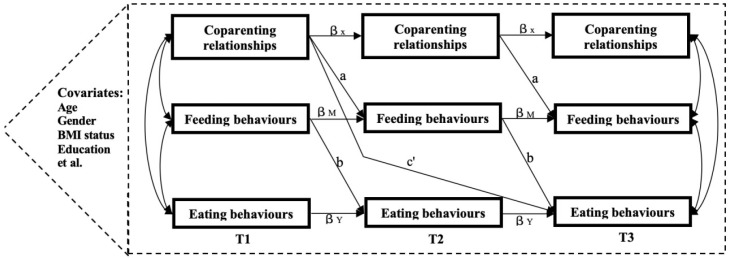
The cross-lagged analysis model with mediating effects of intergenerational co-parenting relationships in this study. Note: T1: December 2020; T2: December 2021; T3: December 2022. a, b = longitudinal mediating effect; c′ = direct effect; β_X_, β_Y_, β_M_ = The effect of time points before and after each variable are also called autoregressive effect.

Multiple imputation was performed using the expectation-maximization (EM) algorithm applied exclusively to core variables—coparenting relationship, feeding behaviors, child eating behaviors (CEBQ), and covariates. We confirmed critical SEM assumptions before estimation across three measurement waves: acceptable multicollinearity (VIFs < 10) and no significant common method bias (Harman’s test < 40% threshold). A cross-lagged longitudinal SEM was conducted to examine both the influence of parent–grandparent coparenting relationships on children’s eating behaviors and the mediating role of caregivers’ feeding behaviors (seen Figure 3). All models systematically incorporated the comprehensive set of covariates, including children’s characteristics (age, gender, temperament), parental characteristics (BMI, education, annual family income), and grandparents’ characteristics (age, education, co-residence status).

We added children’s characteristics (e.g., age, gender, and temperament), parents’ characteristics (e.g., BMI status, education, and annual family income), and grandparents’ characteristics (e.g., age, education, and living together with children or not) as covariates in our models.

Model fit was evaluated by the following indices: comparative fit index (CFI), Tucker–Lewis index (TLI), root mean square error of approximation (RMSEA), standardized root mean-square residual (SRMR), Akaike information criterion (AIC), and Bayesian information criterion (BIC). Model parameters were estimated with a 95% confidence interval (CI). Statistical significance was set at *p* < 0.05 (two-tailed). A model demonstrates good fit if RMSEA and SRMR < 0.08 [36], and GFI, AGFI, and CFI > 0.9 [37,38].

## 3. Results

### 3.1. Baseline Demographic Characteristics of the Sample

In the main urban families, the majority of parents participating in intergenerational coparenting were mothers (77.84%, *n* = 267). The grandparents involved in intergenerational co-parenting were predominantly female, with the following distribution from highest to lowest: grandmothers with 173 (50.44%), maternal grandmothers with 119 (34.69%), grandfathers with 30 (8.75%), and maternal grandfathers with 21 (6.12%). In these nuclear families, there are a total of four different co-parenting combinations, which are mother–grandparents (41.98%, *n* = 144), mother–maternal grandparents (35.86%, *n* = 123), father–grandparents (17.20%, *n* = 59), and father–maternal grandparents (4.96%, *n* = 17). Among them, the proportion of grandparents with an educational level below junior high school was 50.72%, slightly higher than those with a senior high school education or above (49.28%). The children were 184 boys (53.64%) and 159 girls (46.36%), and their ages were between 3.12 and 4.19 years, with an average of 3.71 years (SD = 0.29). Complete demographic characteristics are provided in Supplementary Table S1.

### 3.2. The Characteristics of Parent–Grandparent Coparenting, Caregivers’ Feeding Behaviors and Preschool Children’s Eating Behaviors

Table 1 presents the scores of parent–grandparent coparenting relationships at three-time points in descending order. The scores of positive dimensions (coparenting support, coparenting agreement) were higher than the midpoint (3.0), and the scores of negative dimensions (coparenting undermining, good cop–bad cop) were lower than the midpoint (3.0). In feeding behaviors, only the forced feeding method at T3 is below the midpoint (3.0), while the other six feeding methods are all above the median value of 3.0 at T1–T3. Among the seven preschool children’s eating behaviors, the mean scores of initiative eating were higher than those of the other six child eating behaviors.

### 3.3. The Effects of Parent–Grandparent Coparenting Relationships on Preschool Children’s Eating Behaviors

We conducted a cross-lagged analysis using SEM. Firstly, correlations among three variables (parent–grandparent coparenting relationships, caregivers’ feeding behaviors, and preschool children’s eating behaviors) across three timepoints were examined. The results demonstrated that among these variables were generally consistent, which met the assumptions required for the SEM model (Shown in Supplementary Table S2). Then, we estimated a total of one hundred and forty models adjusting for covariates, of which only six between parent–grandparent coparenting relationships and eating behaviors were found to be prospectively significant via encourage healthy eating. Table 2 details the fitting index results of the six models. After controlling for potential confounding variables and autoregressive effects, the final models were moderate to good fit.

**Figure 4:**
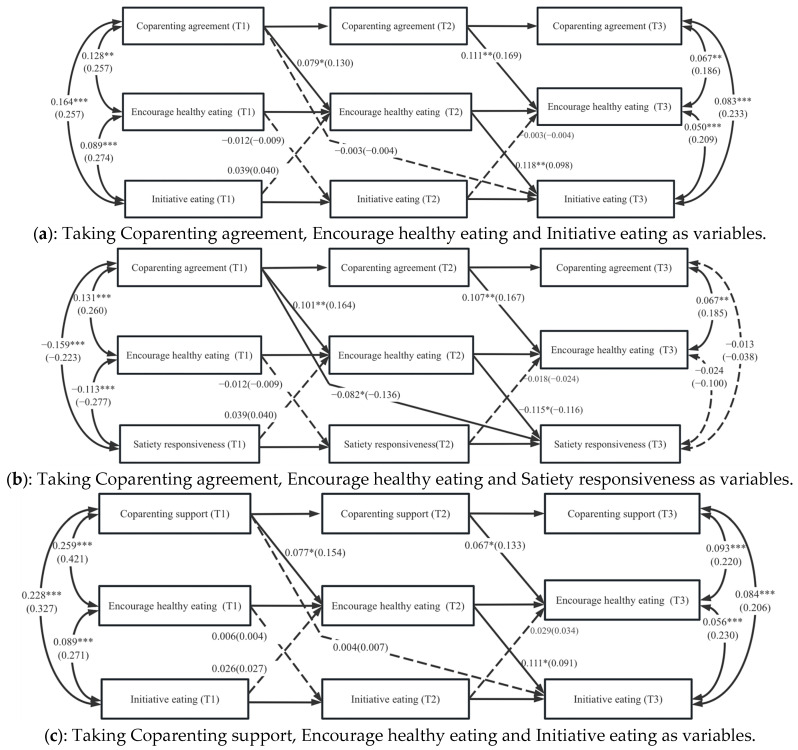

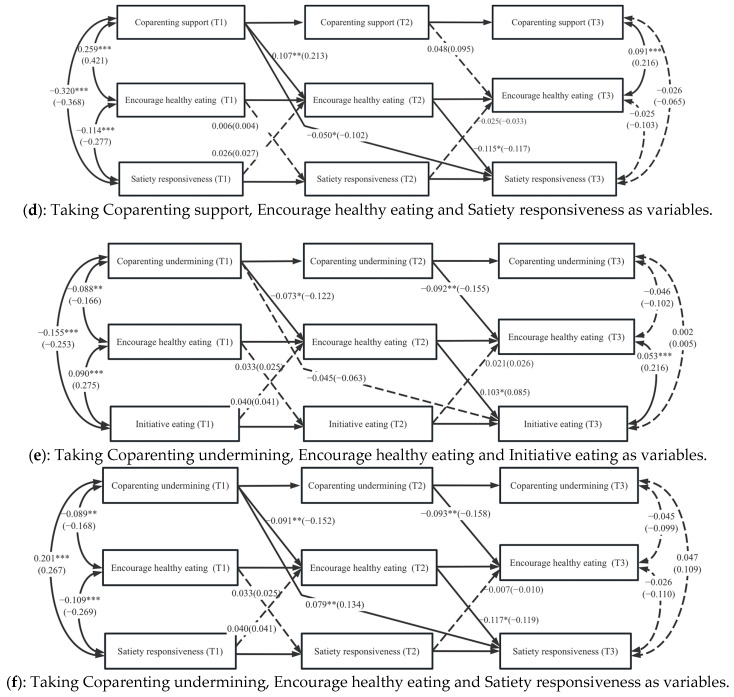
(**a**–**f**). The cross-lagged model with mediating effects of caregivers’ feeding behaviors between parent–grandparent coparenting relationships and preschoolers’ eating behaviors. Note: Full lines represented significant relationships, and dotted lines represented non-significant relationships. The numbers in brackets were standardized coefficients, and the numbers outside brackets were non-standardized coefficients; Estimates are adjusted for demographic characteristics: children’s characteristics (e.g., age, gender, and temperament), parents’ characteristics (e.g., BMI status, education, and annual family income), and grandparents’ characteristics (e.g., age, education, and living together with children or not) and the following variables: preschool children’s temperament and parent depression status. * *p* < 0.05, ** *p* < 0.01, *** *p* < 0.001.

As shown in Figure 4a–f, the mediation analyses examined the extent to which caregivers’ feeding behaviors mediated the associations between intergenerational co-parenting relationships and preschoolers’ eating behaviors in the total sample. In all six models, encouragement of healthy eating served as a significant mediator in three models, demonstrating partial mediation effects in Figure 4b,d,f. As shown in Figure 4b T1 coparenting agreement significantly negatively predicted T3 satiety responsiveness under the mediator role of T2 encourage health eating. In detail, the indirect effect was −0.012, with a 95% confidence interval (CI) ranging from −0.022 to −0.001. Similarly, T1 coparenting support had a significant negatively indirect effect on T3 satiety responsiveness, also partially mediated by T2 encourage healthy eating (seen in Figure 4d). The indirect effect was −0.012, with a 95% confidence interval (CI) ranging from −0.023 to −0.002. Table 3 presents a breakdown of the mediation effects, direct effects, total effects, and the proportion of mediation effects for the seven model’s paths.

The bidirectional associations between caregivers’ feeding behaviors and eating behaviors were significant as depicted in Figure 4d, where there is a mutual predictive effect between caregivers’ feeding behavior and preschool children’s feeding behavior. It manifested as T1 preschool children’s satiety response significantly positively predicted T2 caregivers’ encourage healthy eating (*β* = 0.122~0.151, *SE* = 0.046~0.057, *t* = 2.631~2.664, *p* = 0.008~0.009). Encourage healthy eating at T2 was positively associated with preschool children’s satiety response at T3 (*β* = −0.117~−0.115, *SE* = 0.049~0.050, *t* = −2.336~−2.334, *p* = 0.019~0.020).

## 4. Discussion

This longitudinal study examined how parent–grandparent coparenting relationships influence preschool children’s eating behaviors through parental feeding practices in urban Chinese families. Our findings partially support Hypothesis 1, revealing that intergenerational coparenting indirectly affects children’s eating behaviors, with encouragement of healthy eating emerging as a significant mediator. These findings underscore the cross-temporal mediating role of caregivers’ feeding behaviors between intergenerational co-parenting relationships and preschoolers’ eating behaviors.

The results also provide partial support for Hypothesis 2, demonstrating bidirectional relationships between parental feeding practices and children’s eating behaviors. Consistent with the FST [22], the cross-lagged model demonstrated that preschool children’s satiety responsiveness significantly positively predicted parents’ encouragement of healthy eating. In addition, parents’ encouragement of healthy eating was found to negatively predict subsequent preschool children’s satiety response.

### 4.1. Coparenting Relationships and Child Eating Behaviors

High-quality coparenting relationships facilitate the establishment of consistent family rules, which are associated with improved child psychosocial and health functioning [39]. In our study, we found that the quality of parent–grandparent coparenting relationships was high in urban multigenerational families, characterized by coparenting agreement and support. According to the cross-lagged mediation model, higher levels of coparenting agreement and coparenting support enhance caregivers’ encouragement of healthy eating, which ultimately reduces children’s satiety responsiveness. These findings may be further explained by psychological mechanisms where inconsistent, responsive feeding practices—such as accurately interpreting and respecting children’s satiety cues—help children develop intrinsic self-regulation skills. When caregivers avoid pressuring or restricting eating behaviors, children learn to recognize internal hunger and fullness signals, fostering long-term healthy eating patterns [40]. The quality of parent–grandparent coparenting relationships may amplify this process by modeling cohesive, child-centered feeding strategies across caregivers. Previous researchers, through cross-sectional surveys and observational methods, have demonstrated that positive coparenting relationships are associated with responsive feeding practices [40,41]. According to Belsky’s process model of the determinants of parenting behavior [42], social support from relatives can enhance parental caregiving practices by promoting key aspects of family well-being, such as parents’ mental health and marital functioning, thereby generating positive spillover effects on parenting behavior. In this study, supportive grandparenting behaviors, including financial assistance and practical support such as caring for preschoolers’ dietary needs and school transportation, can enhance parents’ psychological well-being and address their practical needs. This in turn may generate positive spillover effects on parenting behaviors.

Due to the influence of Chinese filial piety culture [42] and the relatively high educational attainment of grandparents in this study, they would actively adopt appropriate methods to avoid conflicts and promote a more harmonious intergenerational co-parenting relationship [43]. This harmonious intergenerational relationship creates a family atmosphere that is more conducive to parents adopting responsive feeding behaviors, thereby helping children develop healthier eating behaviors, such as lower levels of satiety responsiveness.

Intergenerational conflicts in feeding practices emerged as a significant challenge. From the perspective of the normative systems, a normative system of values and beliefs that influence practice or behavior [44]. Because the current food environment differs from that of previous generations, grandparents may be operating within an outdated nutritional framework that includes different dietary rules and definitions of healthy eating [43,44,45]. Intergenerational discrepancies in child-rearing values and practices represent a prevalent challenge in co-parenting dynamics, particularly in multigenerational households. Under traditional Chinese cultural influences, divergent parenting philosophies and grandparents’ preference for plump children inevitably lead to intergenerational coparenting conflicts in child-rearing [46]. When there is a lack of support and a prevalence of conflict between parents and grandparents in the process of joint child-rearing, the caregivers’ attention tends to be focused on their arguments and the negative emotions that arise from these disputes [10]. This, in turn, may adversely affect their individual parenting behaviors towards the child. From the perspective of familial power dynamics, traditional Chinese society accorded greater authority to elders (particularly grandparents) parents, with family interactions structured through a patriarchal hierarchy emphasizing age-based deference and Confucian filial piety norms [47,48]. If there is no clear and reasonable demarcation of boundaries between parents’ authority in child-rearing and grandparents’ responsibilities in child-rearing, it may give rise to intergenerational conflicts and undermining [8]. Results also revealed that coparenting undermining is associated with lower levels of encouragement of healthy eating, which ultimately predicts a higher level of satiety responsiveness. Qualitative studies also indicate that intergenerational conflicts between grandparents and parents hinder the adoption of healthy feeding practices within families [49]. Similarly, in this study, parent–grandparent undermining also significantly and positively predicts children’s unhealthy eating behaviors--satiety responsiveness. The aforementioned findings suggest a potential avenue for future research: intergenerational coparenting relationships may influence children’s eating behaviors through specific caregiving feeding practices.

### 4.2. Bidirectional Feeding–Eating Dynamics

The study revealed important bidirectional relationships between feeding practices and eating behaviors. This study found one child-driven association, which children’s satiety response positively predicted caregivers’ encourage healthy eating. If children exhibit reduced appetite during meals, caregivers may express concern about their child’s nutritional intake and weight regulation. They may be inclined to adopt responsive feeding behaviors, such as encouraging healthy eating practices, to stimulate the child’s interest and curiosity in tasting diverse foods. Jansen et al. also identified a child-driven association in which satiety responsiveness predicted increased maternal structured feeding behaviors between ages 2 and 5 years [50]. Structure feeding behavior is essentially a consistent responsive feeding practice that emphasizes the “reciprocity between child and caregiver” [19,51]. However, Rodgers’ study reported contrasting findings, indicating that children’s satiety responsiveness was positively associated with nonresponsive feeding practices [47]. This discrepancy may be attributed to the differing developmental stages of the children based on their age. Specifically, the participants in Rodgers’ study were two years old, whereas the participants in this study ranged from three to six years of pre-school age. In accordance with Trust models and developmental psychology theories [48,52], as children grow older, their satiety sensitivity—an indicator of effective dietary self-regulation—progressively develops. Caregivers should prioritize responsive feeding behaviors by attentively interpreting the child’s hunger or satiety cues, rather than relying on nonresponsive practices.

Supporting the partial Hypotheses 2 and consistent with prior research [53,54], more caregivers use of encourage healthy eating predicted lower preschool children’s satiety response reported by caregivers one year later. Contrary to the findings of Yuan et al., their study reported that caregivers’ encourage healthy eating positively influenced children’s satiety responsiveness [18]. The attribution for these inconsistent findings may be conceptual differences, namely, the use of different scales to operationalize satiety responsiveness. This discrepancy stems from differences in perspectives: some studies define it as an ability reflecting well-regulated eating competence from the child’s perspective, while others define it as the amount of food consumed by the child as perceived from the parent’s perspective, which manifests as a representation of food intake. In the study conducted by Yuan et al., satiety was defined as satiety sensitivity, that is, fullness sensitivity reflecting good self-regulation in eating capability [18], while in the present study and similar investigations, satiety was operationalized as the child consuming a limited quantity of food in a meal [50,53]. Caregivers may misinterpret behaviors such as leaving food uneaten, rapid satiety, or slow eating as problematic, leading them to pressure the child to consume more, rather than understanding that it may be a response to signals of child’s satiety cues [55].

### 4.3. Methodological Considerations

In the 140 cross-lagged models, we only found six models suggesting that coparenting relationships indirectly affected children’s eating behaviors through caregivers’ encourage healthy eating. All mediating and bidirectional relationships were weak in the study, partly due to the autocorrelations between the three main variables and controls for the covariates (e.g., children’s BMI status, caregivers’ education, and annual family income). The cross-lagged path analysis was applied to explore the chicken-and-egg question because it is a practical statistical approach to explore the causal correlations [56,57]. In current study, the cross-lagged analysis was used to explore the causal correlations between a specific coparenting relationship, feeding practice, and eating behavior, instead of the combined effects of three types of behaviors, which could cause non-significant effects in most models. In the current study, certain co-parenting figures (e.g., maternal grandfathers, paternal grandfathers) had insufficient sample sizes, precluding direct paired comparisons (e.g., mother–grandmother vs. father–grandfather dyads). Critically, our exclusive reliance on parental self-reported data represents a significant methodological limitation. The absence of grandparent perspectives and observational measures increases susceptibility to social desirability bias, particularly in cultural contexts where familial harmony is highly valued. This constraint may have influenced the reporting of intergenerational dynamics and feeding practices. Hence, different intergeneration parenting combinations were not distinguished in this study, which may lead to instability of the coparenting relationship as an antecedent variable.

## 5. Strengths, Limitations, and Future Directions

Despite having several strengths, including the considerably large sample size and the controlling of confounding variables, there were several limitations that were identified and it is hoped that these could be addressed in future research. Firstly, the study did not conduct stratified analyses by gender to explore potential differences in the associations between parental feeding practices and children’s eating behaviors. Given that prior research has demonstrated that parents employ different feeding practices when dealing with children of different genders. Secondly, the survey was conducted in Shanghai, one of the most developed cities in China. When extrapolating the conclusions, differences in regional economic levels should be considered. Additionally, the participants were generally highly educated. This selection bias may further limit the representativeness of the sample. Thirdly, the underrepresentation of specific coparenting dyads (e.g., father and grandfather pairs, *n* = 59) constrained subgroup analyses. This imbalance precluded meaningful comparisons of lineage specific effects on feeding practices. In future studies investigating the impact of grandparents’ involvement in different cultural backgrounds, differentiating between paternal grandparents and maternal grandparents could facilitate a more nuanced examination of the quantity and quality of grandparents’ coparenting involvement, as well as their associations with parental feeding practices and children’s eating behaviors. Lastly, we collected the data only from the views of parents. Any comparisons between the findings of this study and those that examine the relationship from the grandparents’ perspective need to take this generational perception difference into consideration.

## 6. Practical Implications

Our findings highlight grandparents as crucial partners in promoting children’s healthy eating. Nutrition interventions should actively engage grandparents through culturally adapted approaches that acknowledge their distinct caregiving roles, providing them with updated feeding strategies that align with current dietary guidelines. Future programs could consider designing intergenerational components addressing both parent–grandparent collaboration and individual caregiver education. The demonstrated benefits of coparenting harmony suggest that family-based approaches addressing intergenerational relationships may be particularly effective. Community nutrition initiatives should explore integrating multigenerational engagement models, potentially adapting evidence-based strategies from global contexts to local cultural settings. High-quality parent–grandparent coparenting relationships may exert positive influences on children’s growth and development. It is plausible that similar effects may be observed within the context of feeding. Future intervention studies should focus on the needs of grandparents by providing them with strategies and resources to promote healthy eating among their grandchildren.

## 7. Conclusions

This study advances our understanding of how parent–grandparent coparenting relationships influence children’s eating behaviors through parents’ feeding practices. The results emphasize the importance of fostering positive intergenerational coparenting relationships and responsive feeding practices to support children’s healthy eating behaviors development. However, caution is warranted in interpreting these findings. Given the generally weak to moderate effect sizes observed in our statistical models, the generalizability of the results to broader populations, particularly beyond urban Chinese families with relatively high socioeconomic status, may be limited. Future studies can adopt a stratified multistage sampling method to include people from different regions (e.g., rural areas) and educational levels to further improve the representativeness of the sample and ensure the authenticity of the study findings. Due to the limitation of the self-reported information (e.g., being influenced by social expectations as well as personal perceptions and cognitions), future research can explore parent–grandparent coparenting relationships from the perspective of both grandparents and parents.

## Figures and Tables

**Table 1:** The scores of parent–grandparent coparenting, caregivers’ feeding behaviors and preschool children’s eating behaviors at three-time points (*n* = 343).

Variables	T1 [mean (S.D.)]	T2 [mean (S.D.)]	T3 [mean (S.D.)]
Parent–grandparent coparenting relationships			
Coparenting support	3.95 (1.05)	3.99 (1.06)	3.89 (1.01)
Coparenting agreement	3.38 (0.68)	3.40 (0.64)	3.35 (0.61)
Coparenting undermining	1.95 (1.01)	2.04 (1.03)	2.15 (1.11)
Good cop-bad cop	1.76 (1.11)	1.84 (1.15)	1.86 (1.20)
Caregivers’ feeding behaviors			
Supervised feeding	4.11 (0.72)	4.07 (0.71)	4.02 (0.79)
Behavior-restricted feeding	4.11 (0.55)	4.17 (0.56)	4.13 (0.59)
Encourage healthy eating	4.08 (0.44)	4.05 (0.53)	4.05 (0.50)
Content-restricted feeding	3.51 (0.71)	3.65 (0.69)	3.57 (0.69)
Regard food as rewards	3.34 (0.73)	3.41 (0.76)	3.17 (0.77)
Forced feeding	3.23 (0.76)	3.11 (0.78)	2.86 (0.78)
Preschool children’s eating behaviors			
Initiative eating	3.38 (0.53)	3.52 (0.62)	3.64 (0.59)
Food fussiness	3.04 (0.60)	2.88 (0.69)	2.82 (0.61)
Exogenous eating	2.98 (0.56)	2.88 (0.54)	2.83 (0.52)
Satiety responsiveness	2.67 (0.59)	2.67 (0.65)	2.64 (0.60)
Unhealthy eating habits	2.60 (0.74)	2.40 (0.78)	2.28 (0.69)
Food responsiveness	2.49 (0.54)	2.44 (0.56)	2.40 (0.52)
Emotional eating	2.06 (0.54)	1.89 (0.51)	1.86 (0.53)

Notes: T1: December 2020; T2: December 2021; T3: December 2022.

**Table 2:** Fitting index results of the six cross-lagged models (*n* = 343).

Parent–Grandparent Coparenting Relationships	Feeding Behaviors	Eating Behaviors	Figure	*χ* ^2^	*df*	CFI	TLI	RMSEA	SRMR	AIC	BIC
Coparenting agreement	Encourage healthy eating	Initiative eating	Figure 4a	304.163	135	0.870	0.818	0.068	0.087	4649.112	4908.730
		Satiety responsiveness	Figure 4b	145.984	95	0.950	0.925	0.044	0.070	4834.819	5076.408
Coparenting support	Encourage healthy eating	Initiative eating	Figure 4c	299.712	141	0.884	0.837	0.064	0.091	4912.253	5182.688
		Satiety responsiveness	Figure 4d	184.558	100	0.929	0.891	0.056	0.081	5057.353	5313.365
Coparenting undermining	Encourage healthy eating	Initiative eating	Figure 4e	243.070	138	0.902	0.866	0.053	0.073	4913.379	5162.179
		Satiety responsiveness	Figure 4f	172.557	118	0.941	0.914	0.041	0.065	5056.176	5312.187

Notes: CFI, Comparative Fit Index; TLI, Tucker–Lewis index; RMSEA, Root Mean Square Error of Approximation; SRMR, Standardized Root Mean-square Residual; AIC, Akaike Information Criterion; BIC, Bayesian Information Criterion.

**Table 3:** Indirect effects and 95% confidence intervals for the mediation model (*n* = 343).

Model Pathway	Type of Effect	Estimated (*β*)	SE	Boot 95%CI
Coparenting agreement T1 → Encourage healthy eating T2 → Initiative eating T3 Figure 4a	Total effect	0.006	0.034	[−0.050, 0.062]
	Indirect effect	0.009	0.006	[0.000, 0.019]
	Direct effect	−0.003	0.034	[−0.060, 0.053]
	The proportion of indirect effect	33.3%		
Coparenting agreement T1 → Encourage healthy eating T2 → Satiety responsiveness T3 Figure 4b	Total effect	−0.094	0.032	[−0.146, −0.041]
	Indirect effect	−0.012	0.006	[−0.022, −0.001]
	Direct	−0.082	0.033	[−0.136, −0.028]
	The proportion of indirect effect	12.8%		
Coparenting support T1 → Encourage healthy eating T2 → Initiative eating T3 Figure 4c	Total effect	0.013	0.027	[−0.032, 0.058]
	Indirect effect	0.009	0.005	[0.000, 0.017]
	Direct	0.004	0.028	[−0.042, 0.050]
	The proportion of indirect effect	69.20%		
Coparenting support T1 → Encourage healthy eating T2 → Satiety responsiveness T3 Figure 4d	Total effect	−0.062	0.027	[−0.107, −0.018]
	Indirect effect	−0.012	0.006	[−0.023, −0.002]
	Direct	−0.050	0.028	[−0.097, −0.003]
	The proportion of indirect effect	19.40%		
Coparenting undermining T1 → Encourage healthy eating T2 → Initiative eating T3 Figure 4e	Total effect	−0.053	0.029	[−0.101, −0.005]
	Indirect effect	−0.007	0.005	[−0.016, 0.001]
	Direct	−0.045	0.030	[−0.094, 0.003]
	The proportion of indirect effect	13.2%		
Coparenting undermining T1 → Encourage healthy eating T2 → Satiety responsiveness T3 Figure 4f	Total effect	0.090	0.030	[0.040, 0.139]
	Indirect effect	0.011	0.006	[0.001, 0.020]
	Direct effect	0.079	0.030	[0.029, 0.129]
	The proportion of indirect effect	12.2%		

## Data Availability

The raw data supporting the conclusions of this article will be made available by the authors on request.

## Supplementary Materials

The following supporting information can be downloaded at: https://www.mdpi.com/article/10.3390/nu17182961/s1, Table S1: Demographic characteristics of the sample; Table S2: Descriptive statistics and correlation coefficients between parent-grandparent coparenting and preschool.
