# Supplementary material for: The Longitudinal Influence of Parent–Grandparent Coparenting Relationships on Preschoolers’ Eating Behaviors in Chinese Urban Families: The Mediating Roles of Caregivers’ Feeding Behaviors
Source: Nutrients. 2025 Sep 15;17(18):2961. doi: 10.3390/nu17182961 (PMC12472853; doi:10.3390/nu17182961)
Supplement: Supplementary file 1 [file nutrients-17-02961-s001.zip › supplementary Table S1.pdf]

Table S1. Demographic characteristics of the sample (n = 343).

	Variables	Characteristics	*n* (%)/mean (S.D.)
**Children**	Age (year)	Range from 3.12 to 4.19	3.71 (0.29)
	Gender	Male	184 (53.64)
		Female	159 (46.36)
	BAZ status	Underweight	2 (0.58)
		Normal weight	294 (85.71)
		Overweight	33 (9.62)
		Obese	14 (4.08)
	Single child	Yes	224 (65.31)
		No	119 (34.69)
	Duration of breastfeeding	<6	86 (25.07)
	(month)	6 ~12	168 (48.98)
		>12	89 (25.95)
	Temperament	Anger/frustration	4.20 (0.81)
		Inhibition control	5.20 (0.75)
**Parents**	Mother's age (year)	Range from 26.00 to 49.00	34.33 (3.83)
	Father's age (year)	Range from 27.00 to 50.00	36.06 (4.41)
	Mother's BMI status	Underweight	36 (10.50)
		Normal weight	245 (71.43)
		Overweight	52 (15.16)
		Obese	10 (2.92)
	Father's BMI status	Underweight	9 (2.62)
		normal weight	162 (47.23)
		Overweight	125 (36.44)
		Obese	47 (13.70)
	Mother's education	Junior high school	7(2.04)

		Senior high school	21 (6.12)
		Junior college or above	315 (91.84)
	Father's education	Junior high school	9 (2.62)
		Senior high school	19 (5.54)
		Junior college or above	315 (91.84)
	Annual family income (CNY)	99,999 or below	5 (1.46)
		100,000–199,999	50 (14.58)
		200,000–299,999	88 (25.66)
		300,000–399,999	71 (20.70)
		400,000–499,999	57 (16.62)
		500,000 or above	72 (20.99)
**Grandparents**	Age (year)	Range from 49.00 to 76.00	61.67 (5.22)
	BMI status	Underweight	9 (2.62)
		normal weight	206 (60.06)
		Overweight	112 (32.65)
		Obese	16 (4.66)
	Education	Primary school or below	60 (17.49)
		Junior high school	114 (33.24)
		Senior high school	146 (42.57)
		Junior college or above	23 (6.71)
	Living together with children	Yes	225 (65.60)
		No	118 (34.40)

Notes: S.D., standard deviation; BMI, body mass index; BAZ, BMI-for-age Z score.
